# A Case of Chorea Illustrating Its Neuropathic Heredity

**Published:** 1901-03-16

**Authors:** 


### A CASE OF CHOREA ILLUSTRATING ITS NEUROPATHIC HEREDITY.

Dr. Philip Meirowitz, of the New York Post-graduate Medical School,[1] publishes an instructive case with clinical history and a genealogical table to illustrate the importance of neuropathic heredity in determining the appearance of chorea. The case is that of a boy aged six years whose mother wished to administer tincture of iron for his failing appetite. By mistake she gave the boy ten minims of tincture of iodine in a wineglass of water. The child at once said, "Mamma, this don't taste like the iron." The mother thereupon seizing the boy ran in great fright to the nearest druggist, who administered some starch and water. The boy was intensely agitated and cried incessantly: "O mamma, am I going to die?" By degrees he became calm, but next day he began to twitch and jerk his limbs and mouth. Facial and ocular movements of choreic nature set in. Of the limbs the right arm showed the greatest disturbances of movement. There was also present some paresis of the arms, and on attempting to flex the fingers or the knee some pain was felt. There was no muscular tenderness. The pulse was 96 per minute and regular; the heart's action was normal. Inquiry into his family history revealed the following facts, which are grouped into a genealogical table:—

Paternal Grandmother: had Rheumatism, and an insane brother.

Maternal Grandfather: Nervous.

Father has rheumatism, and has a sister with rheumatism.

Mother has fainting spells and morbid fears.

Uncle insane.

Uncle has chorea.

Patient has chorea, and has had rheumatism at the age of five years.

The case shows not only the intimate relation existing between chorea and the rheumatic taint, but illustrates the fact that both of these are sequelæ prone to manifest themselves as expressions of a deeper and more fundamental neuropathic heredity condition—a condition to which French observers have applied the term "neuro-arthritic diathesis."

[1] The Post-graduate, Jan., 1901.

